# Antidotal treatment of botulism in rats by continuous infusion with 3,4-diaminopyridine

**DOI:** 10.1186/s10020-022-00487-4

**Published:** 2022-06-03

**Authors:** James B. Machamer, Edwin J. Vazquez-Cintron, Sean W. O’Brien, Kyle E. Kelly, Amber C. Altvater, Kathleen T. Pagarigan, Parker B. Dubee, Celinia A. Ondeck, Patrick M. McNutt

**Affiliations:** 1 U.S. Army Medical Research Institute of Chemical Defense, Gunpowder, MD 21010 USA; 2 BASF, Research Triangle, Durham, NC 27709 USA; 3 Wake Forest Institute for Regenerative Medicine, Wake Forest School of Medicine, Winston-Salem, NC 27101 USA

**Keywords:** Botulinum neurotoxin, Botulism, 3,4-diaminopyridine, Neuromuscular junction, Endplate recordings, Preclinical models, Drug delivery

## Abstract

Botulinum neurotoxins (BoNTs) are highly potent, select agent toxins that inhibit neurotransmitter release at motor nerve terminals, causing muscle paralysis and death by asphyxiation. Other than post-exposure prophylaxis with antitoxin, the only treatment option for symptomatic botulism is intubation and supportive care until recovery, which can require weeks or longer. In previous studies, we reported the FDA-approved drug 3,4-diaminopyridine (3,4-DAP) reverses early botulism symptoms and prolongs survival in lethally intoxicated mice. However, the symptomatic benefits of 3,4-DAP are limited by its rapid clearance. Here we investigated whether 3,4-DAP could sustain symptomatic benefits throughout the full course of respiratory paralysis in lethally intoxicated rats. First, we confirmed serial injections of 3,4-DAP stabilized toxic signs and prolonged survival in rats challenged with 2.5 LD_50_ BoNT/A. Rebound of toxic signs and death occurred within hours after the final 3,4-DAP treatment, consistent with the short half-life of 3,4-DAP in rats. Based on these data, we next investigated whether the therapeutic benefits of 3,4-DAP could be sustained throughout the course of botulism by continuous infusion. To ensure administration of 3,4-DAP at clinically relevant doses, three infusion dose rates (0.5, 1.0 and 1.5 mg/kg∙h) were identified that produced steady-state serum levels of 3,4-DAP consistent with clinical dosing. We then compared dose-dependent effects of 3,4-DAP on toxic signs and survival in rats intoxicated with 2.5 LD_50_ BoNT/A. In contrast to saline vehicle, which resulted in 100% mortality, infusion of 3,4-DAP at ≥ 1.0 mg/kg∙h from 1 to 14 d after intoxication produced 94.4% survival and full resolution of toxic signs, without rebound of toxic signs after infusion was stopped. In contrast, withdrawal of 3,4-DAP infusion at 5 d resulted in re-emergence of toxic sign and death within 12 h, confirming antidotal outcomes require sustained 3,4-DAP treatment for longer than 5 d after intoxication. We exploited this novel survival model of lethal botulism to explore neurophysiological parameters of diaphragm paralysis and recovery. While neurotransmission was nearly eliminated at 5 d, neurotransmission was significantly improved at 21 d in 3,4-DAP-infused survivors, although still depressed compared to naïve rats. 3,4-DAP is the first small molecule to reverse systemic paralysis and promote survival in animal models of botulism, thereby meeting a critical treatment need that is not addressed by post-exposure prophylaxis with conventional antitoxin. These data contribute to a growing body of evidence supporting the use of 3,4-DAP to treat clinical botulism.

**Supplementary Information:**

The online version contains supplementary material available at 10.1186/s10020-022-00487-4.

## Introduction

Botulinum neurotoxins (BoNTs) are highly potent protein poisons produced by the *Clostridium* genus of anaerobic bacteria (Pirazzini et al. 2017). The active neurotoxin is a heterodimer composed of a 100 kDa heavy chain (HC) and 50 kDa light chain (LC) which remain attached through electrostatic interactions and a single disulfide bond. The HC mediates selective binding to endosomal receptors on the presynaptic membrane of peripheral neurons (Dong et al. 2006; Dong et al. 2003). Following neuronal uptake via synaptic endocytosis, the LC translocates into the nerve terminal, where it specifically cleaves neuronal SNARE (soluble *N*-ethylmaleimide-sensitive factor attachment protein receptor) proteins essential for neurotransmitter release (Simpson 2004). Cleavage of SNARE proteins blocks assembly of the core vesicle fusion complex, preventing vesicle fusion and acetylcholine release (Montal 2009; Burgen et al. 1949). As the concentration of cleaved SNARE proteins increases, motor nerve terminals become unable to reliably elicit muscle contraction, causing muscle weakness that progresses to flaccid paralysis. Clinical symptoms of botulism emerge 12–36 h after exposure to BoNT, resulting from peripheral blockade of neurotransmission at neuromuscular junctions and autonomic nerve terminals (Sobel 2005). At lethal doses, neuroparalytic symptoms originally manifest as cranial nerve dysfunctions that rapidly advance to respiratory arrest (Lindstrom and Korkeala 2006).

The only specific treatment for botulism is passive immunization with immunoglobulin antitoxin, which blocks neuronal uptake of BoNT but has no effect on toxin molecules already internalized into neurons (Al-Saleem et al. 2011). Antitoxin is highly effective when administered prior to neuronal uptake (Richardson 2019; Yu et al. 2017). However, BoNT exhibits both a high affinity for presynaptic receptors and a delayed manifestation of paralysis; thus, at lethal doses, paralytic quantities of toxin can internalize into motor neurons before symptoms of botulism become apparent (Simpson 2013). Accordingly, administration of antitoxin to symptomatic patients has reduced ability to prevent respiratory paralysis. Indeed, approximately 70% of botulism cases require mechanical ventilation when antitoxin is administered more than 48 h after symptoms appear (Richardson 2019; Yu et al. 2017). In these cases, the principle benefit of delayed antitoxin treatment is to shorten the duration of paralysis by preventing additional neuronal uptake of BoNT. The timely administration of antitoxin is made more challenging by the difficulty in diagnosing early-stages of botulism, the need to request release of antitoxin from regional stockpiles, and the requirement to infuse antitoxin over hours to monitor for hypersensitivity, serum sickness or other adverse infusion reactions (Sobel 2005).

The stark limitations of antitoxin treatment have necessitated a search for anti-botulism therapies that reverse disease in symptomatic patients. Considerable effort has been directed to antidotal treatments, with an emphasis on small molecule inhibitors that block LC metalloprotease activity within the nerve terminal (Duplantier et al. 2016; Pirazzini and Rossetto 2017). However, development of small molecule LC inhibitors is complicated by multiple factors, including a large substrate-enzyme interface, extensive conformational flexibility, requirement for multiple molecules to block the structurally diverse toxin serotypes, rapid clearance, and need to inhibit intracellular targets without associated toxicity (Breidenbach and Brunger 2004; Silvaggi et al. 2007; Kumar et al. 2014; Li et al. 2010; Eubanks et al. 2007). Intraneuronal delivery of therapeutic antibodies was recently reported to have antidotal efficacy in mice and non-human primates (McNutt, et al. 2021; Miyashita et al. 2021); however, recovery from neuromuscular paralysis still requires the regeneration of intact SNARE proteins (Bartels et al. 1994). Consequently, independent of the development of antidotal therapies, there remains a critical need for fast-acting treatments to sustain respiration until definitive care can be administered.

Without proper medical care, foodborne botulism is lethal in approximately 60% of cases (Dembek et al. 2007; O'Horo et al. 2017). The combination of antitoxin plus intensive care support has reduced mortality to ~ 5%, albeit at substantial cost (O'Horo et al. 2017). Since even moderate-scale outbreaks can cause devastating effects on local healthcare resources, BoNTs are considered Tier 1 select agents with high risk of use as a mass casualty bioweapon (Centers for Disease Control and Prevention 2017). Of the eight BoNT serotypes, BoNT serotype A (BoNT/A) is responsible for half of foodborne botulism cases in the United States (National Botulism Surveillance 2022a). The same properties that make BoNT/A a terror threat (extraordinary potency, neuronal specificity and long duration of action) also render it a highly effective cosmetic and therapeutic drug, leading to its widespread use as the active component in most neurotoxin-based pharmaceuticals (Pirazzini et al. 2017; Brashear 2008; Foran et al. 2003). Given the dangers of botulism poisoning from bioterror attack, foodborne exposure and medical mis-use, identifying therapies for BoNT/A poisoning is a high priority.

Oral amifampridine (3,4-diaminopyridine phosphate) is FDA-approved for treatment for Lambert Eaton Myasthenic Syndrome (LEMS) in adults. LEMS is an autoimmune disease caused by reduced acetylcholine release and neuromuscular weakness (McEvoy et al. 1989). Mechanistically, 3,4-diaminopyridine (3,4-DAP) prolongs action potential duration by reversibly blocking voltage-gated potassium channels, facilitating presynaptic Ca^2+^ influx and increasing acetylcholine release (Thomsen and Wilson 1983; Kirsch and Narahashi 1978; Ojala et al. 2021). In LEMS patients, 3,4-DAP partially reverses neuromuscular weakness by restoring threshold release of acetylcholine, with a median effective serum level of 30 ng/mL (Thakkar et al. 2017). Since botulism symptoms also result from reduced acetylcholine release (Bradford et al. 2018), we and others have hypothesized 3,4-DAP may attenuate botulism symptoms (Kim et al. 1984; Siegel et al. 1986; Mayorov et al. 2010; Beske et al. 2017). Indeed, 3,4-DAP reverses muscle paralysis in isolated mouse diaphragms poisoned by multiple BoNT serotypes, with particularly efficacy in treatment of BoNT/A (Bradford et al. 2018; Siegel et al. 1986). Administration of 3,4-DAP transiently improved respiratory function and prolonged survival in mice challenged with 5 LD_50_ BoNT/A, confirming symptomatic efficacy in vivo (Vazquez-Cintron et al. 2020). However, these studies revealed 3,4-DAP’s symptomatic benefits are limited by its rapid clearance. Furthermore, because of the fast distribution and short half-life of 3,4-DAP, it was not possible to establish serum levels at which symptomatic improvements emerged.

To address these limitations, we developed a continuous 3,4-DAP infusion model in rats and measured dose-dependent effects on toxic signs and survival after lethal challenge with BoNT/A. We found 3,4-DAP infusion reverses toxic signs and has antidotal effects at serum levels consistent with clinical dosing. Therapeutic benefits did not involve direct antagonism of BoNT molecular toxicity and required continuous infusion for between 4 and 13 days, suggesting antidotal outcomes emerged from sustained therapeutic benefits. 3,4-DAP is the first small molecule to reverse systemic paralysis and promote survival in animal models of botulism, meeting a critical treatment need that is not addressed by post-exposure prophylaxis with conventional antitoxin.

## Methods

### Animals

Male Sprague–Dawley rats (350–425 g; Charles River Laboratories) were group-housed, maintained on a 12 h diurnal cycle and provided a standard diet with regular enrichment and water ad libitum. Rats were implanted with subcutaneous catheters (Charles River Laboratories) prior to delivery via incision in the upper back and insertion of the catheter into a dorsal tunnel, with the infusion port secured between the scapulae. Catheters were flushed every 3–5 d with 0.5 mL saline to ensure patency. For intoxication, rats were randomly assigned to groups and administered BoNT/A diluted in buffered saline with 0.2% gelatin in a volume of 250 µL by tail vein injection. Rats were euthanized with 3% isoflurane followed by decapitation.

### Determination of BoNT/A intravenous LD_50_ in rats

Botulinum neurotoxin serotype A (10 µg/mL) was purchased from Metabiologics (Madison, WI, USA) and stored at 4 °C. BoNT/A was diluted to working concentrations in PBS with 0.2% gelatin (ThermoFisher, Waltham, MA). The rat intravenous LD_50_ was determined using a stagewise, adaptive design for potency testing (Feder et al. 1991), in which rats were administered 88 pg/kg (n = 2), 136 pg/kg (n = 2), 168 pg/kg (n = 4), 184 pg/kg (n = 5), 208 pg/kg (n = 4), 228 pg/kg (n = 2) or 276 pg/kg (n = 2) BoNT/A. Survival was recorded at daily intervals over 7 d and the intravenous LD_50_ was determined to be 175.2 pg/kg (95% CI: 158.8–186.0 pg/kg) by simple logistical regression (Additional file 1: Fig. S1A).

### 3,4-DAP pharmacokinetic analysis

To determine steady-state concentration (C_SS_), rats were infused through subcutaneous catheters at a constant rate of 333 μL/h. The surgically implanted infusion port was stabilized with a rat harness (VAH95AB, Instech Laboratories, Plymouth Meeting, PA, USA) to prevent accidental removal and connected to a KVAH95T tether kit, which ran from the infusion port through a MCLA counterbalance level arm (Instech Laboratories) to 10 mL plastic syringes (Becton Dickinson, Franklin Lakes, NJ, USA) containing saline vehicle or 3,4-DAP prepared in saline. Syringes were controlled with a Pump 11 Elite syringe pump (Harvard Apparatus, Holliston, MA, USA). Tethers were filled with treatment solution prior to connection to the infusion port.

To measure C_SS_, 3,4-diaminopyridine (3,4-DAP; Millipore Sigma, MO, USA) was diluted in saline and infused for 24 h at dose rates of 0.36 mg/kg∙h, 0.72 mg/kg∙h and 1.5 mg/kg∙h. Saline vehicle infusions were used as controls. Rats were monitored for adverse effects throughout each infusion. Immediately before stopping infusions, rats were briefly anesthetized with isoflurane and 0.5 mL blood was collected from the tail vein. After blood collection, rats were perfused with saline for 3 h to clear residual 3,4-DAP from the infusion tubing. Rats were infused using escalating doses, with intervening 5 d washouts.

Blood was allowed to clot for 30 min, centrifuged at 2000 x*g* for 15 min at 4 °C and sera were aliquoted and stored at − 80 °C until analyzed. 3,4-DAP levels were measured by liquid chromatography—tandem mass spectrometry using an Agilent 1290 Infinity liquid chromatograph (Agilent Technologies, Santa Clara, CA) with an Ultra Silica 3 μm column (Restek, Bellefonte, PA, USA) and a Sciex 6500 QTrap triple quadrupole mass spectrometer (Sciex, Ottawa, CA, USA). The calibration curve was linear over the concentration range of 0.39–400 ng/mL (r^2^ = 0.999) with significant association between predicted and measured concentrations (*p* < 0.0001 versus zero-slope). The relationship between 3,4-DAP infusion dose rate and C_SS_ was determined via linear regression.

### In vitro measurements of 3,4-DAP inhibition of LC/A

Dose-dependent effects of 3,4-DAP on BoNT proteolytic activity were measured using the Botest FRET assay (BioSentinel, Inc) according to manufacturer’s directions. Briefly, BoNT/A (100 pM), 10 mM DTT and 0.01, 0.1, 1, 10 or 100 µM 3,4-DAP were added to a 96-well clear-bottom black plate (Greiner) and incubated with the SNAP-25 FRET substrate at 37 °C in a fluorescent plate reader (Molecular Devices). After 1 h, reactions were excited at 434 nm and emissions were measured at 470 nm and 526 nm. Proteolytic activity of the light chain was quantified by dividing the relative fluorescence unit (RFU) value at 526 nm by the RFU value at 470 nm. SNAP-25 FRET substrate alone and with 100 µM 3,4-DAP were used as negative controls to confirm there was no spontaneous FRET cleavage and that 3,4-DAP did not interfere with the assay. 100 nM BoNT/A was used as a positive control for substrate cleavage.

### 3,4-DAP efficacy studies

For serial injection studies, rats were weighed and intoxicated by intravenous administration of 2.5 LD_50_ BoNT/A (440 pg/kg). Starting 32 h after intoxication, rats were given 15 subcutaneous injection of 2 mg/kg 3,4-DAP or saline at 1.5 h intervals and monitored for toxic signs and survival every 1.5 h. For continuous infusion studies, rats were acclimated to the infusion harness starting 1 d before intoxication. On the day of intoxication, rats were weighed and intoxicated by tail-vein injection of 440 pg/kg of BoNT/A. At 27 h after intoxication, infusion of saline or 3,4-DAP was started at 333 µL/h as described above. Saline and 3,4-DAP infusates were prepared daily using the weights measured at time of intoxication. To replace infusates, the infusion pump was stopped for less than 2 min so depleted syringes could be replaced with syringes containing fresh infusate. Toxic signs and mortality were recorded at ≤ 6 h intervals from 0 to 5 d and at daily intervals thereafter. To mitigate malnutrition, oral gavage of liquid nutrition (Boost Very Vanilla complete nutritional drink; Nestlé Health Science, Vevey, Switzerland) was administered twice daily through a ball-tip stainless steel feeding needle (Cadence Science, Staunton, VA, USA) for a total daily administration of 5 mL/kg. Oral gavage volumes were calculated using the weights measured at time of intoxication. If rats drank from the feeding needle prior to gavage, gavage was not performed. Oral gavage started no earlier than 6 d and continued until voluntary consumption of food and water was observed (no later than 11 d). Infusion was stopped at 14 d and infusion harnesses were removed. Clinical observations were continued for an additional 7 d before euthanasia at 21 d. In one study, infusion was stopped at 5 d and rats were euthanized immediately or monitored for toxic signs and survival at 3 h intervals.

### Semi-quantitative assessment of toxic signs

Three researchers blinded to the groups conducted clinical scoring of toxic signs of botulism using the following scoring rubric (modified from 39): (A) respiratory signs: mild abdominal paradox (score of 1), moderate abdominal paradox (Dong et al. 2003), or severe abdominal paradox and/or agonal respiratory pattern (Burgen et al. 1949); and (B) skeletomuscular signs: salivation (Pirazzini et al. 2017) and lethargy (Pirazzini et al. 2017), limb weakness (Dong et al. 2003), or total body paralysis (lack of righting reflex, 6). Animals scoring 12 during consecutive observations were euthanized and animals were given a score of 16 upon death.

For safety studies of 3,4-DAP, healthy rats were evaluated for salivation, altered gait/limb weakness and central nervous effects using the Racine scale (Luttjohann et al. 2009). Assessments were conducted at 30 min intervals after single injections, at 30 min after each injection during a treatment series, and at 6 h intervals during continuous infusions.

### Endplate recordings

Endplate recordings were conducted as previously described (Vazquez-Cintron et al. 2020). Briefly, rats were deeply anesthetized with isoflurane and euthanized by decapitation. Diaphragm and associated phrenic nerves were isolated and pinned on a Sylgard dissection dish in oxygenated Tyrode’s solution (in mM: 137 NaCl, 5 KCl, 1.8 CaCl_2_, 1 MgSO_4_, 24 NaHCO_3_, 1 NaH_2_PO_4_ and 11 d-glucose, pH 7.4) at 22–24 °C. To confirm hemidiaphragm integrity, phrenic nerves were stimulated by a bipolar stimulating electrode (FHC, Inc, Bowdoin, ME, USA) using 0.2 ms square wave pulses with increasing amplitudes (Digitimer, Fort Lauderdale, FL, USA) until muscle contraction was observed. Muscle contraction was then selectively blocked by 30 min incubation with the muscle-specific, voltage-gated sodium channel blocker, μ-conotoxin GIIIB (1–2 μM, Alamone Labs, Israel) (Cruz et al. 1985). Recordings were performed with a Heka EPC10 patch clamp amplifier and sharp glass electrodes (10–20 MΩ) pulled with a Sutter Instrument P1000 (Novato, CA, USA). Muscles fibers were impaled close to endplate junctions, and recordings with starting resting membrane potential (RMP) of less than − 60 mV were discarded. To eliminate residual effects of 3,4-DAP infusion, hemidiaphragm preparations were incubated with regular washes until the decline in endplate potential (EPP) amplitudes stabilized (approximately 120 min) before collecting electrophysiological data. In pilot studies, a similar incubation had no effect on neurotransmission parameters in naïve hemidiaphragms.

For each muscle fiber, 10 EPPs were elicited at 0.2 Hz stimulation followed by a 2 min recording of miniature endplate potentials (mEPPs). Diaphragms were maintained for no more than 3 h with regular exchanges of oxygenated Tyrode’s solution. EPP amplitudes were averaged for each muscle fiber and corrected for non-linear summation (McLachlan and Martin 1981). mEPPs were detected with event detection software (version 1.7.0, Axograph Scientific, San Luis Obispo, CA, USA) and averaged for each muscle fiber. A total of 20 endplates were recorded per each hemidiaphragm. Recordings that produced no EPPs or mEPPs were discarded. Quantal content (QC) was calculated by dividing EPP amplitude by mEPP amplitude. For intoxicated tissues that produced EPPs without mEPPs, QC was calculated using the average mEPP amplitude among endplate recordings for each hemidiaphragm.

### Statistics

Efficacy data represents the cumulative results from five separate studies (n = 8 rats per study) with mixed doses and 1–4 vehicle rats in each study. Continuous variables with normal distribution are presented as mean ± SEM and toxic sign scores are presented as median values or median ± interquartile range (IQR). Toxin potency was determined using simple logistical regression. Median survival was determined from Kaplan–Meier survival curves and compared among all treatment groups using Mantel-Cox log-rank test. Pairwise comparisons in median survival time were made against vehicle controls using a Bonferroni-adjusted significance threshold, which was determined by dividing 0.05 by the number of pairwise comparisons. Progression of toxic signs was compared among groups using two-way repeated measures ANOVA with Tukey’s multiple comparisons test. Survival outcomes among three or more groups were first compared using the Chi-square test followed by pairwise comparisons against vehicle controls with Fisher’s exact test using a Bonferroni-adjusted significance threshold. EPP success rates were compared using Kruskal Wallis test by ranks, while QC, mEPP amplitudes and mEPP frequency were compared using one-way ANOVA with Tukey’s multiple comparisons test. Because transmitter release varied more among endplates from a single hemidiaphragm than among diaphragms within a treatment group, the sample size (n) was defined as the number of endplates in each condition. Significant differences among treatment conditions were sustained when average endplate values per animal (n = 3–8 per group) were used instead of individual endplate data. Statistical comparisons were made in Prism version 9.0 (Graphpad Software, San Diego, CA). Differences were considered significant at the 95% confidence level (P < 0.05) except as described above. Additional details on statistical comparisons, normality tests and sample sizes are presented in Additional file 5: Table S2, individual figure legends and the results section.

## Results

### Bolus treatment with 3,4-DAP transiently reverses symptoms and prolongs survival in rats challenged with 2.5 LD_50_ BoNT/A

**Fig. 1:**
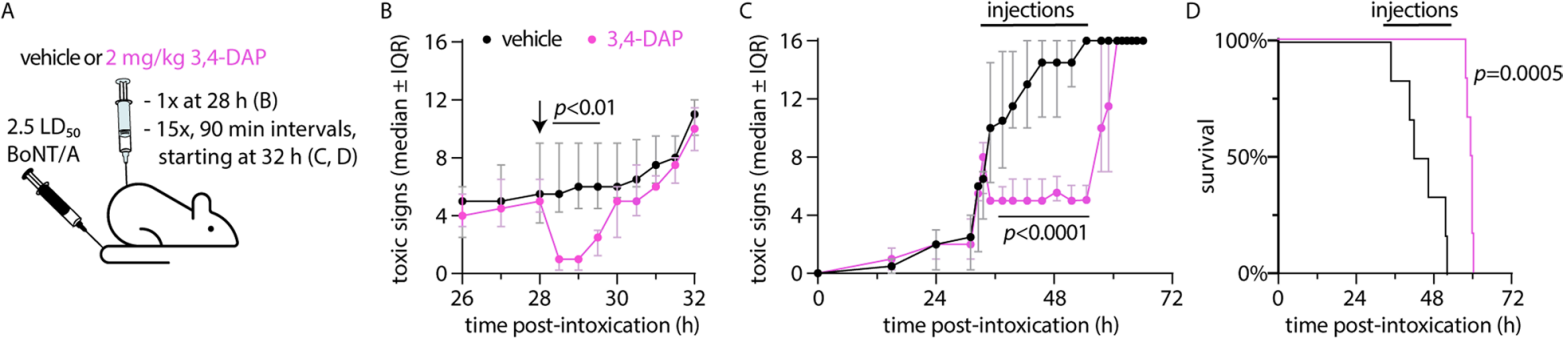
Bolus administration of 2 mg/kg 3,4-DAP reverses clinical signs of botulism and prolongs survival. **A** Summary of experimental models. **B** Median ± interquartile ratio (IQR) toxic signs for rats given a single injection of vehicle (black) or 3,4-DAP (pink) at 28 h after challenge with 0.44 ng/kg BoNT/A (2.5 LD_50_ BoNT/A; n = 4 per group). Toxic signs were monitored at 30 min intervals. Arrow indicates time of injection. (C, D) Median ± IQR toxic signs (**C**) and survival curves (**D**) for rats given 15 injections of vehicle (black) or 3,4-DAP (pink) at 90 min intervals, starting 32 h after challenge with 2.5 LD_50_ BoNT/A (n = 6 per group). Black lines above graphs indicate treatment period during which injections were administered. Specific details on *n*-values and statistical comparisons for this and subsequent figures are presented in Additional file 5: Table S2

To evaluate 3,4-DAP treatment in a lethal rat botulism model, we first determined no-adverse effect levels for 3,4-DAP doses in healthy rats. Safety studies confirmed single administration of up to 8 mg/kg 3,4-DAP dose was well-tolerated, with minor neurological toxicity emerging at 16 mg/kg 3,4-DAP (Additional file 4: Table S1). Previous studies have shown 2 mg/kg 3,4-DAP prolongs survival in mice challenged with 1.5–5.0 LD_50_ BoNT/A (Vazquez-Cintron et al. 2020) and produces relatively similar Cmax values in rats (1.1 µg/mL) (Ishida 2019) and mice (0.8 µg/mL) (Vazquez-Cintron et al. 2020). Based on these data, bolus injection studies were conducted at 2 mg/kg 3,4-DAP. Because previous studies in mice indicated 3,4-DAP had a transient effect on botulism symptoms (Vazquez-Cintron et al. 2020), we next determined the pharmacodynamic duration of 3,4-DAP treatment in rats intoxicated with 0.44 ng/kg BoNT/A (2.5 LD_50_; Fig. 1A, B). Rats were given a single injection of vehicle or 2 mg/kg 3,4-DAP at 28 h after intoxication, when they exhibited moderate toxic signs of botulism (Additional file 1: Fig. S1B). Compared to vehicle, which had no apparent effect, 3,4-DAP treatment reduced toxic signs within 30 min (Fig. 1A, B). This effect proved transient and toxic signs rebounded between 90 and 120 min after 3,4-DAP treatment.

We next tested whether repeated injections of 3,4-DAP prolonged survival versus vehicle in rats challenged with 2.5 LD_50_ BoNT/A. Natural history studies revealed the median survival time at this challenge dose in control rats was 41 h (range: 16–56 h; Additional file 1: Fig. S1C). Power analysis indicated fifteen treatments, given at 90 min intervals starting 32 h after intoxication, would be sufficient to determine whether 3,4-DAP extended median survival (Fig. 1A). This dosing regimen was well-tolerated in healthy rats (Additional file 4: Table S1). The lack of acute toxicity despite the large cumulative 3,4-DAP dose (30 mg/kg given over 22.5 h) can likely be attributed to the short serum half-life of 3,4-DAP in rats (15.9 ± 3.1 min) (Ishida 2019), which corresponds to 5.7 half-lives between 3,4-DAP injections.

At the start of repeated injections, rats displayed abdominal paradox, dysphagia and limb weakness (Fig. 1A, C). Toxic signs continue to progress in the saline-treatment group and 100% of rats were deceased prior to the final treatment, with a median survival time of 13.9 h after the first injection (Fig. 1D). In 3,4-DAP-treated rats, toxin signs improved within 30 min of the first injection and remained stable throughout the injection series. As of 1.5 h after the final injection, 100% of 3,4-DAP treated rats remained alive compared to 0% of vehicle-treated rats (*p* = 0.002). Toxic signs worsened by 3 h after the final 3,4-DAP injection and all rats were deceased within 6 h (Fig. 1C, D). These data confirmed 3,4-DAP had robust, albeit transient, beneficial effects on toxic signs at moderate stages of disease in lethally challenged rats. Furthermore, they indicated continued 3,4-DAP treatment significantly extended survival beyond the time when vehicle-treated rats were deceased.

### 3,4-DAP does not directly antagonize BoNT/A light chain proteolytic activity

3,4-DAP increases acetylcholine release from intoxicated motor nerve terminals, providing a physiological mechanism for reversal of paralysis (Bradford et al. 2018; Vazquez-Cintron et al. 2020). However, it remained possible that 3,4-DAP therapeutic effects included disrupting BoNT/A light chain (LC/A) proteolytic activity. To test this hypothesis, LC/A activity was quantified in the presence of 0–100 µM 3,4-DAP using a FRET-based SNAP-25 cleavage assay (Additional file 2: Fig. S2). LC/A cleavage of SNAP-25 was not inhibited up to 100 µM 3,4-DAP, which is tenfold higher than peak muscle tissue concentrations in rats after administration of 2 mg/kg 3,4-DAP (Ishida 2019). These data suggest 3,4-DAP reversal of toxic signs did not involve LC/A inhibition.

### Continuous infusion of 3,4-DAP has antidotal effects

**Fig. 2:**
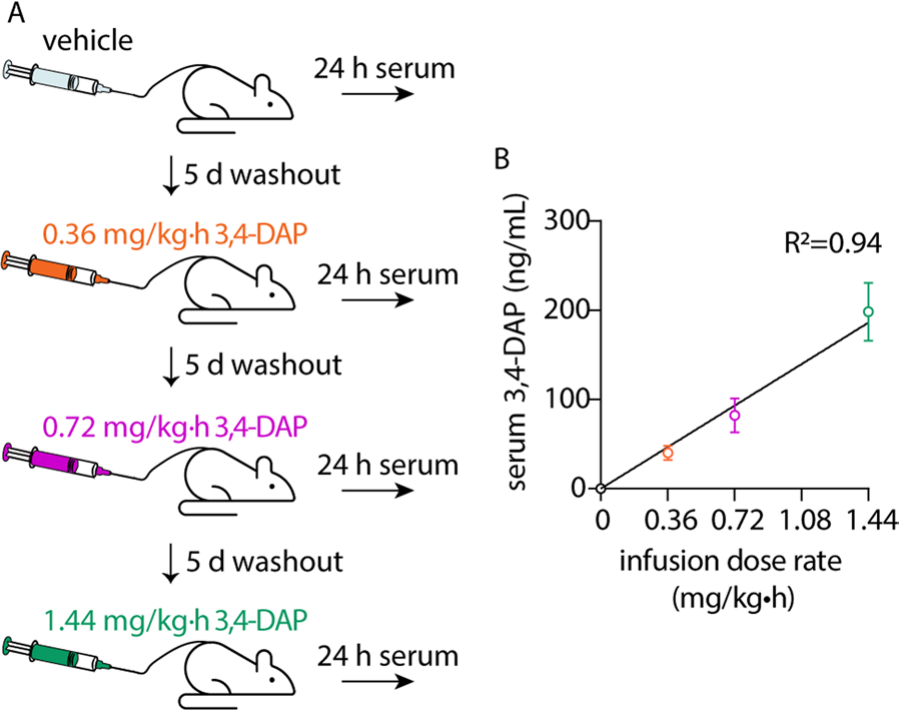
Correlation of infusion dose and 3,4-DAP C_SS_ after 24 h. Catheterized rats (n = 7) were subcutaneously infused for 24 h with 3,4-DAP at 0 (saline vehicle), 0.36, 0.72 and 1.44 mg/kg h, with a 5-day washout between doses. Serum was collected after 24 h and analyzed for 3,4-DAP. **A** Summary of experimental strategy. **B** Mean ± SD values for serum 3,4-DAP concentrations after 24 h infusion at each infusion dose rate. Linear regression demonstrated a strong fit between serum 3,4-DAP levels and infusion dose rate (R^2^ = 0.94)

Given the ability of repeated 3,4-DAP injections to prolong survival during the acute onset of botulism, we next asked whether continuous administration of 3,4-DAP could sustain survival throughout the course of disease. Although the duration of respiratory paralysis is unknown in rodents, the median duration of artificial ventilation during clinical botulism is 1.5–2 weeks (Richardson 2019; Yu et al. 2017). Consequently, we developed a subcutaneous infusion model in which 3,4-DAP could be continuously delivered to intoxicated rats for indefinite durations. To determine the relationship between 3,4-DAP infusion dose rate and steady-state serum concentrations (C_SS_), C_SS_ was determined in sera from healthy rats following 24 h of continuous infusion at 0.36 mg/kg∙h, 0.72 mg/kg∙h or 1.44 mg/kg∙h 3,4-DAP (Fig. 2A). Although C_SS_ was expected to stabilize within 90 min, infusions were continued for 24 h to assess neurological toxicity. 3,4-DAP C_SS_ followed first-order kinetics (R^2^ = 0.94), without evidence of saturation effects over 24 h (Fig. 2B; Additional file 5: Table S2). Behavioral signs of neurological toxicity were not observed at any infusion dose (Additional file 4: Table S1).

**Fig. 3:**
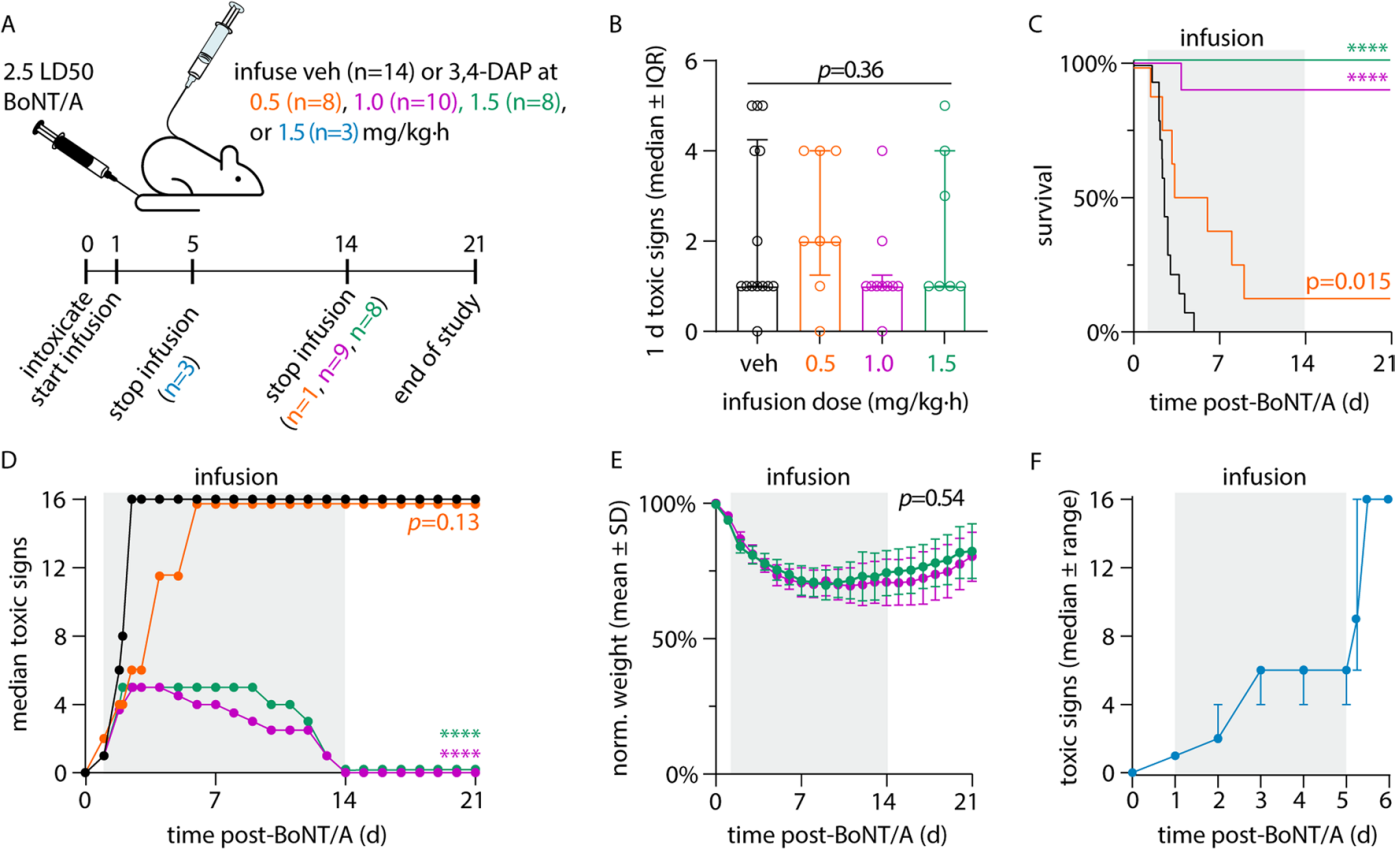
Continuous infusion of 3,4-DAP has both symptomatic and antidotal effects in lethally intoxicated rats. Catheterized rats were intoxicated by intravenous injection of 2.5 LD_50_ BoNT/A. At 27 h after intoxication, continuous infusion was started with saline vehicle (n = 14) or 3,4-DAP at 0.5 mg/kg∙h (n = 8), 1.0 mg/kg∙h (n = 10) or 1.5 mg/kg∙h (n = 8). Toxic signs, weight and survival were monitored at 6–24 h intervals. Colors are consistent among panels A-E. Gray boxes represent infusion period. **A** Summary of experimental strategy. **B** Toxic signs at start of infusion. Data are presented as median ± IQR. **C** Kaplan–Meier survival curves for each treatment group. **D** Median toxic signs for each group over time. For panels **C** and **D**, significance indicators show pairwise comparisons made to vehicle. **E** Mean ± SD normalized body weights for survivors in 1.0 mg/kg∙h and 1.5 mg/kg∙h treatment groups. **F** Median ± IQR toxic signs for rats (n = 3) infused from 1 to 5 d with 1.5 mg/kg∙h 3,4-DAP. Treatment was withdrawn at 5 d and toxic signs were monitored at 6 h intervals. ****p < 0.0001, *p < 0.05

To test the effects of continuous infusion of 3,4-DAP on botulism symptoms, rats were lethally intoxicated with 2.5 LD_50_ BoNT/A and randomized into treatment or vehicle groups (Fig. 3A). Intoxicated rats were infused with saline vehicle (333 µL/h) or 3,4-DAP at 0.5 mg/kg∙h (target C_SS_ = 65 ng/mL), 1.0 mg/kg∙h (target C_SS_ = 130 ng/mL) or 1.5 mg/kg∙h (target C_SS_ = 195 ng/mL). These three infusion dose rates were chosen to align with clinical serum levels reported after oral dosing with 3,4-DAP (6.5–218 ng/mL) (Thakkar et al. 2017). The target C_SS_ also exceeded the median effective dose for treatment of LEMS (30 ng/mL) (Thakkar et al. 2017). Infusion of 3,4-DAP started at 27 h and continued through 5 d or 14 d and rats were monitored for rebound of botulism symptoms for up to 7 d after infusion was stopped.

3,4-DAP infusion had a strong dose-dependent effect on both toxic signs and survival. At start of infusion, 92.5% (37/40) of rats exhibited toxic signs of botulism (*p* = 0.36 among groups; Fig. 3B). Toxic signs continued to progress in vehicle-treated rats, causing 100% mortality with a median survival time of 2.5 d (range 2.0–4.5 d; Fig. 3C). Infusion with 1.0 mg/kg∙h 3,4-DAP improved survival to 90% (*p* < 0.0001 versus vehicle) while infusion with 1.5 mg/kg∙h 3,4-DAP improved survival rate to 100% (*p* < 0.0001 versus vehicle; Fig. 3C). In surviving rats, toxic signs stabilized by 2 d as moderate abdominal paradox, limb weakness and salivation, and began to resolve after 5 d (1.5 mg/kg∙h) or 9 d (1.0 mg/kg∙h; Fig. 3D). There was no difference in toxic signs at the two highest infusion dose rates (*p* = 0.92, Additional file 5: Table S2), although there was a trend towards earlier recovery at 1.5 mg/kg∙h versus 1.0 mg/kg∙h. Surviving rats remained active, alert and responsive after infusion was stopped at 14 d after intoxication, without rebound of botulism symptoms (Fig. 3D; Additional file 3: Video S1) and with progressive weight recovery over the subsequent week (Fig. 3E). In comparison, infusion with 0.5 mg/kg∙h 3,4-DAP had partial efficacy, extending the median survival time versus vehicle (4.7 d vs 2.5 d; *p* = 0.015) without affecting survival rates (1/8 survival; *p* = 0.36 versus vehicle).

Because toxic signs began to improve by 5 d in the 1.5 mg/kg∙h group, we were curious whether 3,4-DAP treatment was needed beyond that point. To address this question, BoNT/A-intoxicated rats were infused at 1.5 mg/kg∙h 3,4-DAP from 1–5 d, after which infusion was stopped and rats were monitored for toxic signs and survival. Toxic signs worsened within 6 h after 3,4-DAP withdrawal and all rats were deceased within 12 h, indicating that spontaneous recovery had not progressed enough to allow for survival without 3,4-DAP treatment (Fig. 3F). Collectively, these data suggest antidotal efficacy of 3,4-DAP emerges from sustained therapeutic benefits.

### Characterization of neuromuscular transmission during intoxication and recovery

We previously reported that diaphragm neuromuscular transmission is profoundly reduced at terminal stages of botulism in mice (Vazquez-Cintron et al. 2020). The 3,4-DAP infusion model described here provides a unique opportunity to characterize changes in diaphragm neurophysiology throughout the course of disease. Because 3,4-DAP can be washed out of isolated diaphragms, this model also allowed characterization of basal levels of neurotransmission at intoxicated motor nerve terminals at times when untreated rats would be deceased.

To conduct a preliminary analysis of diaphragm intoxication and recovery after lethal BoNT/A challenge, intracellular recordings were used to measure nerve-elicited endplate potentials (EPPs) and spontaneous miniature endplate potentials (mEPPs) in diaphragms isolated from four cohorts: (a) naïve rats, (b) intoxicated rats infused with 1.5 mg/kg∙h 3,4-DAP and sacrificed at 5 d, when toxic signs were maximal; (c) intoxicated rats infused with 1.0 mg/kg∙h 3,4-DAP from 1–14 d and sacrificed at 21 d; and (d) intoxicated rats infused with 1.5 mg/kg∙h 3,4-DAP from 1–14 d and sacrificed at 21 d.

**Fig. 4:**
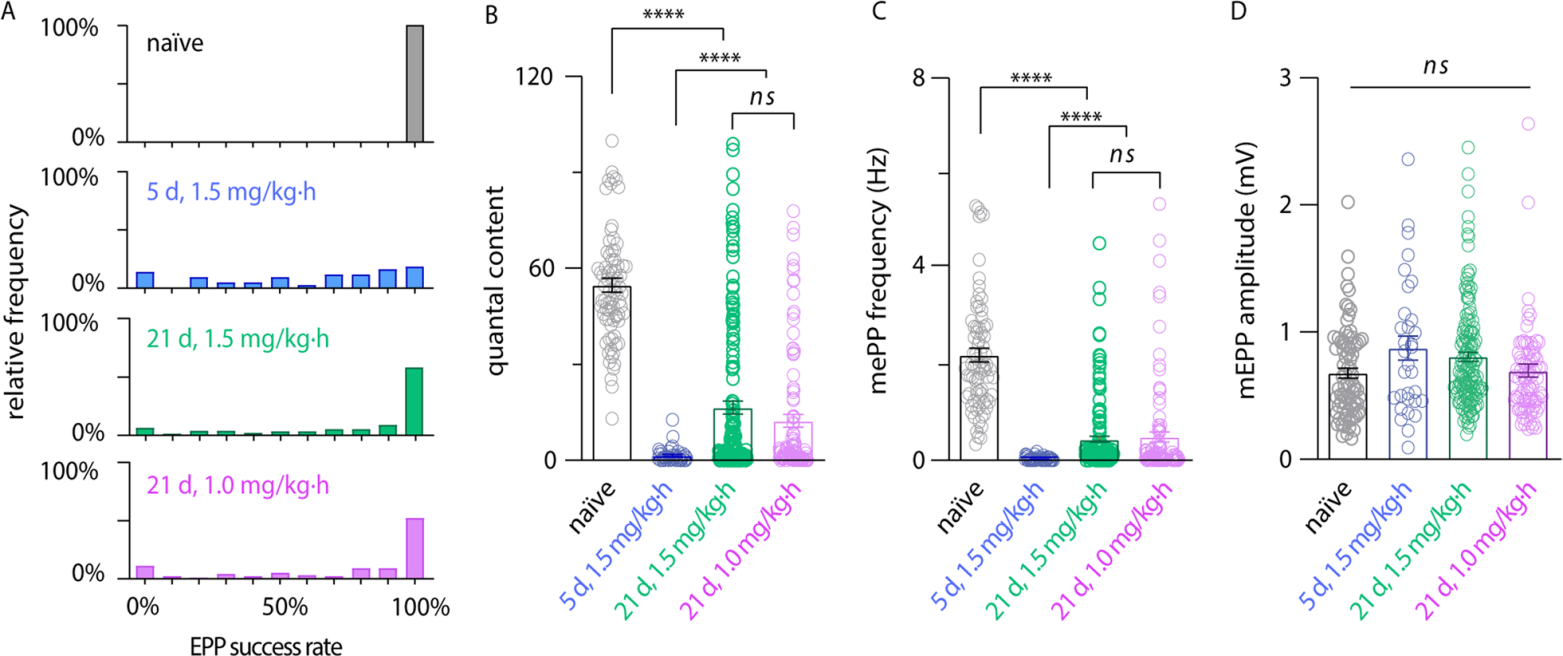
Time-dependent recovery of diaphragm endplate potentials (EPPs) in 3,4-DAP infused intoxicated rats. Diaphragm endplate success rates, quantal content (QC) and miniature EPPs (mEPPs) were compared among naïve rats (black); rats intoxicated with 0.44 ng/kg BoNT/A, infused with 3,4-DAP from 1 to 14 d at 1.0 mg/kg∙h (green) or 1.5 mg/kg∙h (pink) and euthanized at 21 d; or rats intoxicated with 0.44 ng/kg BoNT/A, infused with 1.5 mg/kg∙h 3,4-DAP from 1 to 5 d and euthanized (blue). **A** Histogram of EPP success rates. During each endplate recording, phrenic nerves were stimulated with a train of 10 impulses at 0.2 Hz and the percentage of impulses that produced EPPs were determined. **B**–**E** Mean ± SEM scatter plots for (**B**) Quantal content, (**C**) mEPP frequencies and (**D**) mEPP amplitudes. For **B** and **D**, each circle represents average responses of 10 stimulations in one endplate. For **C**, each circle represents mEPP frequencies measured in one endplate. ****p < 0.0001, *ns* indicates significant

We first characterized diaphragm neurotransmission in 3,4-DAP infused rats at 5 d after BoNT/A intoxication, when survival is dependent on 3,4-DAP infusion (Fig. 3F). To characterize basal neurotransmission levels without contamination by residual 3,4-DAP, 5 d diaphragm preparations were washed until EPPs stabilized (approximately 120 min) before collecting endplate recordings. EPP success rates were measured by stimulating phrenic nerves with 10 nerve impulses at 0.2 Hz and quantifying the fraction of stimuli that produced EPPs. While naïve diaphragms produced EPPs with 100% success rate, 5 d diaphragms had a median EPP success rate of 70% (95% CI[50%, 80%], *p* < 0.0001 vs naïve), with a distribution ranging from 0 to 100% (Fig. 4A). Consistent with profound diaphragm paralysis, quantal content (QC), which approximates the number of synaptic vesicles that fuse during a nerve action potential (Del Castillo & Katz, 1954), was reduced by 97.2% at 5 d (53.7 vesicles versus 1.5 vesicles, *p* < 0.0001; Fig. 4B). Similarly, mEPP frequency was reduced by 99.2% at 5 d compared to naïve endplates (2.15 ± 0.13 Hz versus 0.017 ± 0.003 Hz, *p* < 0.0001; Fig. 4C). mEPP frequency represents the stochastic fusion of synaptic vesicles at individual release sites (Rotshenker and Rahamimoff 1970). Because mEPP frequency is directly correlated to the availability of functional (i.e., non-intoxicated) release complexes, decreased mEPP frequency in BoNT-treated tissues is an agnostic marker of synaptic intoxication (Bradford et al. 2018; Beske et al. 2016). mEPP amplitudes, which are not affected by BoNT intoxication (Bradford et al. 2018), were measured as a quality control check. mEPP amplitudes were not different between naïve and 5 d diaphragms (0.68 ± 0.04 versus 0.87 ± 0.09; *p* = 0.38), suggesting recording conditions were consistent among tissues (Fig. 4D). Collectively these data indicate neuromuscular reliability and acetylcholine release are profoundly decreased at 5 d, providing a mechanism for respiratory failure and death after treatment withdrawal.

We also measured neurotransmission in survivors to characterize the degree of recovery from 5 to 21 d. At 21 d, median EPP success rates were recovered to 100% in both the 1.0 mg/kg∙h infusion group (95% CI[90%, 100%]; *p* < 0.0001 vs 5 d) and in the 1.5 mg/kg∙h infusion group (95% CI[100%, 100%]; *p* < 0.0001 vs 5 d; Fig. 4A). Interestingly, whereas QC and mEPP frequency were significantly improved by 21 d, both parameters remained depressed compared to naïve diaphragms (Fig. 4B, C). The 1.0 mg/kg∙h and 1.5 mg/kg∙h treatment groups were indistinguishable in success rate, mEPP frequency and QC (*p* > 0.99 for each parameter), indicating that 3,4-DAP infusion from 1 to 14 d did not have residual dose-dependent effects at 21 d. mEPP amplitudes were not affected by treatment (*p* = 0.62). Persistent evidence of neuromuscular impairment at 21 d is consistent with clinical reports indicating ventilatory recovery from botulism can require months (Wilcox et al. 1990). Although the minimum level of phrenic neurotransmission required for survival has yet to be determined, comparisons of 5 d and 21 d diaphragm neurophysiologies illustrate the profound and long-lasting effects BoNT has on neuromuscular function.

## Discussion

Botulism paralysis is caused by reduction of acetylcholine release from motor nerve terminals to subthreshold levels required for muscle contraction. 3,4-DAP acutely reverses this pathophysiology by increasing the amount of acetylcholine released during neuronal action potentials (Bradford et al. 2018). Here we demonstrate 3,4-DAP has dose-dependent symptomatic and antidotal efficacy in rats challenged with 2.5 LD_50_ BoNT/A. We first determined that single administration of 2 mg/kg 3,4-DAP reversed toxic signs for approximately 90 min, while repeated injections led to sustained symptomatic improvement and prolonged survival throughout the treatment period. To compensate for the short therapeutic benefit of bolus 3,4-DAP treatments, we developed a continuous infusion system and found 3,4-DAP enables robust survival in a dose-dependent fashion. Rats remained asymptomatic after treatment withdrawal at 14 d after intoxication, despite neurophysiological evidence of residual impairment at diaphragm motor endplates. Therapeutic benefits could not be attributed to LC/A inhibition and required continuous infusion beyond 5 d and up to 14 days after intoxication, suggesting antidotal outcomes require sustained 3,4-DAP treatment until botulism paralysis naturally recedes. Antidotal outcomes emerged at target C_SS_ values of 130 ng/mL, which is consistent with standard clinical dosing levels (Thakkar et al. 2017). Collectively, these data demonstrate continuous infusion of 3,4-DAP produces rapid and sustained therapeutic benefits that can allow survival from lethal challenge with BoNT/A. 3,4-DAP is the first small-molecule therapy to directly reverse toxic signs and promote survival when administered post-symptomatically after a lethal botulism challenge.

In vitro mechanistic studies (Bradford et al. 2018; Beske et al. 2017) and animal efficacy studies (Siegel et al. 1986; Vazquez-Cintron et al. 2020) indicate 3,4-DAP therapeutic benefits are influenced by BoNT serotype, dose and stage of disease. The therapeutic mode of action of 3,4-DAP provides a mechanistic basis for this conditional efficacy. Neuromuscular junctions contain thousands of synaptic vesicle release sites, a small fraction of which (~ 5–10%) probabilistically fuse and release acetylcholine during each action potential (Tarr et al. 2013; Ruiz et al. 2011; Laghaei et al. 2018). The molecular process of vesicle fusion is mediated by the amino- to carboxy-terminal zippering of SNARE motifs from the neuronal SNARE proteins SNAP-25, Syb and Stx, which initiates membrane fusion and acetylcholine release (Poirier et al. 1998; Sutton et al. 1998; Hanson et al. 1997). BoNT light chains specifically cleave these SNARE proteins, damaging or eliminating the SNARE motifs and interfering with assembly of the fusogenic SNARE complex. Consequently, BoNT intoxication leads to the graded reduction in EPP amplitudes as release sites become progressively disrupted by cleaved SNARE proteins (Bradford et al. 2018; Beske et al. 2017; Lu 2015; Hayashi et al. 1994; Khounlo et al. 2017). Once EPPs drops below threshold levels, nerve action potential fail to elicit muscle contraction (Wood and Slater 2001). 3,4-DAP partially counteracts BoNT intoxication by increasing the number of release sites activated (Bradford et al. 2018; Ng et al. 2017), with clinical benefits emerging once threshold levels of acetylcholine are restored. Accordingly, 3,4-DAP is expected to be most effective in partially intoxicated nerve terminals containing a sufficient population of  intact release sites to support threshold release in response to increased release probability, such as following low-dose intoxications, during the onset of paralysis, and during recovery from paralysis. (Bradford et al. 2018). Comparatively, therapeutic benefits of 3,4-DAP are likely to be smaller after a high-dose exposure, in which few release sites are expected to remain fusogneic due to the high neuronal toxin load. However, in these circumstances, 3,4-DAP treatment may prolong respiratory function during the onset of paralysis until definitive care can be applied.

3,-DAP has enhanced efficacy in treatment of BoNT/A botulism. Whereas other BoNT serotypes destroy the ability of cleaved SNARE proteins to support vesicle fusion (with the possible exception of BoNT/C) (Capogna et al. 1997), BoNT/A-cleaved SNAP-25 remains capable of forming a fusogenic SNARE complex, albeit with reduced efficiency (Lu 2015; Khounlo et al. 2017). Indeed, 3,4-DAP restores synaptic function to cultured neurons and phrenic nerve-diaphragm preparations poisoned by high doses of BoNT/A, but not other serotypes, (Bradford et al. 2018; Beske et al. 2017, 2018; Vazquez-Cintron, et al. 2020). We have attribute this serotype-specific effect to the ability of 3,4-DAP to prolong neuronal depolarization, thereby extending the temporal window during which SNARE-mediated fusion can occur (Beske et al. 2017). Thus, 3,4-DAP has potential to restore neurotransmission in BoNT/A-intoxicated nerve terminals through two processes: (1) increasing the number of release sites activated during an action potential and (2) specifically restoring fusogenic capability to BoNT/A-cleaved SNAP-25. It is unknown whether both mechanisms are required for the antidotal effects observed here. Additional studies are underway to clarify whether antidotal benefits extend to other serotypes, or if  3,4-DAP is less effective in treating non-BoNT/A botulism.

The therapeutic mode of action also identifies a potential limitation of 3,4-DAP in treating high-dose botulism. If release probability is increased excessively, exhaustion of fusion-competent release sites could paradoxically cause synaptic fade and enhance neurotransmission failure, thus confounding therapeutic benefit. While this phenomenon has not been described in the context of botulism, high concentrations of aminopyridines have been reported to enhance synaptic fade during stimulation trains in naïve endplates (Thomsen and Wilson 1983; Ng et al. 2017). Thus, 3,4-DAP may have an inherent limitation in the ability to restore threshold release in severely intoxicated nerve terminals, when few release sites are available to mediate neurotransmitter release. The use of routine neurodiagnostic assays should allow the functional evaluation of cumulative toxicity in botulism patients, allowing clinicians to balance therapeutic efficacy against cumulative toxicity, if necessary.

Although an oral formulation of 3,4-DAP approved for Lambert-Eaton Myasthenic Syndrome (LEMS) has many optimal pharmaceutical properties, including rapid absorption, > 93% bioavailability and < 20% bound to plasma proteins (Amifampridine Drug Approval Package 2022), the short half-life of 3,4-DAP (~ 2.5 h) causes trough effects that results in symptomatic rebound in some LEMS patients (Thakkar et al. 2017). The use of higher doses to prolong the therapeutic window can cause minor but distressing neurological effects, such as paresthesia and tremor (Aisen et al. 1995). Thus, dosing strategies are clinically adjusted to balance the risk of dose-dependent neurological effects against symptomatic benefit. Here, we found the short half-life of 3,4-DAP in rodents (~ 15 min) produced an ephemeral effect on botulism symptoms in vivo. To compensate for the rapid clearance of 3,4-DAP, we developed an infusion model to test continuous 3,4-DAP treatment on botulism symptoms while avoiding the troughs and peaks of bolus administration. Although we were initially concerned the hysteresis between intracellular persistence and plasma persistence caused by ion trapping (Kirsch and Narahashi 1978) would necessitate a high Cmax to drive neuronal uptake of therapeutic quantities of 3,4-DAP, near-complete survival occurred at steady-state serum levels that were ~ 10% of Cmax produced by bolus administration of 2 mg/kg 3,4-DAP (Ishida 2019), indicating that therapeutic benefit did not require a loading dose. Furthermore, there was no evidence of functional toxicity or apparent loss of efficacy throughout the infusion period. These data suggest that continuous infusion of 3,4-DAP can sustain symptomatic benefits in treatment of botulism and, by extensions, LEMS, allowing administration of a wider range of doses while reducing the risk of adverse neurological effects and symptomatic breakthrough.

Previous efforts to understand the toxic mechanisms of BoNT on neurotransmission have relied on either supraphysiological intoxication of isolated diaphragm muscle preparations (Dolly et al. 1987) or local administration of paralytic doses to skeletal muscles (Kim et al. 1984), neither of which recapitulate the toxicokinetics of a systemic lethal challenge. The antidotal effects of infused 3,4-DAP allowed us to study intoxication and recovery of phrenic neurotransmission after lethal BoNT/A challenge for the first time. Endplate recordings revealed profound acute impairment of diaphragm neurotransmission at 5 d after intoxication that is only partially reversed at 21 d. Although robust feedback loops can sustain neuromuscular function under conditions of reduced neurotransmission (Wood and Slater 2001; Ermilov et al. 2007), the extent of residual impairment at 21 d was surprising considering surviving rats were bright, alert and responsive. Indeed, hysteresis between depressed neurotransmission and physiological recovery has been observed in local paralysis models (Frick et al. 2007; Adler et al. 2001), suggesting compensatory plasticity is a common response to chronically impaired neurotransmission. Alternatively, endplate recordings may underestimate in vivo neurotransmission for multiple reasons. First, recordings were performed at room temperature and there is a well-established positive correlation between temperature and presynaptic release parameters (Kriebel et al. 1976). Second, rat phrenic nerves discharge in 20–200 Hz bursts during inspiration (Marchenko et al. 2012; Ghali 2018), producing a frequency-dependent facilitation that does not occur at the 0.2 Hz stimulation frequencies used in ex vivo studies (Jackman and Regehr 2017). Given the stimulatory effects of presynaptic Ca^2+^ influx on neurotransmission in BoNT/A-intoxicated nerve terminals (Bradford et al. 2018; Beske et al. 2017, 2018), it is likely that physiological stimulation frequencies would facilitate neurotransmission more than isolated stimuli. Finally, during a typical action potential, motor neurons release four–sixfold more acetylcholine than is necessary to elicit muscle contraction (Wood and Slater 2001). This ‘safety factor’ allows substantial decreases in acetylcholine release without reducing neuromuscular reliability, contributing to the apparent hysteresis between physiology and neurophysiological recordings. This is the first animal model in which changes in neurotransmission in response to a lethal, systemic botulism challenge can be functionally correlated to physiological metrics during recovery, and we anticipate future studies will characterize compensative responses to reversible impairment of phrenic activity.

Although antitoxin typically does not prevent respiratory failure when given to symptomatic patients, it can decrease the duration of paralysis by blocking further uptake of BoNT (Richardson 2019; Yu et al. 2017). Because 3,4-DAP efficacy is inversely related to the extent of intoxication, symptomatic treatment with 3,4-DAP is expected to be more effective in reversing botulism symptoms in patients treated concomitantly with antitoxin. Furthermore, since 3,4-DAP works orthogonally to antitoxin, the risks of adverse drug interactions are low. In comparison to monotherapy, multimodal therapy with antitoxin and 3,4-DAP is likely to accelerate recovery from botulism, reduce the risk of life-threatening hospital-acquired diseases, decrease treatment costs and free limited resources for other critical patients (Arnon et al. 2006; Souayah et al. 2012; Anderson et al. 2019). Furthermore, the majority of botulism patients continue to exhibit neurological deficits and muscle weakness after discharge (Yu et al. 2017; Gottlieb et al. 2007). If these persistent symptoms are related to chronically depressed neurotransmission (as suggested by Fig. 4), then they may also be amenable to 3,4-DAP treatment. Finally, inclusion of a treatment to inhibit or clear LC/A from the nerve terminal would comprise a comprehensive anti-botulism therapy for all aspects of disease.

One limitation of this study is the use of a static dosing model, in which 3,4-DAP infusion doses were determined from rat weights at intoxication. Rats lost up to 25% of body weight over the first week after intoxication, potentially altering the relationship between infusion dose rate and C_SS_. However, signs of 3,4-DAP toxicity were not observed, suggesting putative changes in C_SS_ were well-tolerated. More detailed studies will be required to determine the potential effects of intoxication and physiological stress on 3,4-DAP pharmacokinetic properties. Another limitation is that 3,4-DAP efficacy was evaluated against BoNT/A, whereas foodborne botulism is also associated with serotypes B, E and F (National Botulism Surveillance 2022b). As described above, symptomatic benefits may be less robust for other serotypes. Furthermore, although foodborne botulism cases usually involve less than 5 LD_50_, in rare cases exposures can exceed 100 LD_50_ (Pirazzini et al. 2017). Because 3,4-DAP is less effective in more severely paralyzed nerve terminal, symptomatic benefits may be limited at higher BoNT doses.

In conclusion, BoNT is a highly dangerous select agent with limited treatment options once symptoms emerge. Infusion with the clinically approved drug 3,4-diaminopyridine (3,4-DAP) produces symptomatic and antidotal effects at clinically relevant blood levels in rats lethally challenged with the most common BoNT serotype. Therapeutic benefits emerge at 3,4-DAP exposure levels consistent with approved clinical dosing. Survival requires continuous 3,4-DAP infusion for longer than 4 d, suggesting antidotal outcomes emerged from sustained therapeutic benefits. 3,4-DAP is the first small molecule to reverse systemic paralysis and promote survival in animal models of botulism, showing potential to meet a critical treatment need not addressed by conventional antitoxin. These data illustrate strong translational potential for 3,4-DAP as a treatment of clinical botulism caused by the serotype most commonly associated with human disease.

## Supplementary Information

**Additional file 1: Figure S1. **BoNT/A potency determination and disease progression at 2.5 LD_50_ in rats. (A) Determination of rat intravenous LD_50_. Rats were administered 88–276 pg/kg BoNT/A by tail vein injection and monitored for survival at 24 h intervals through 7 d. Surviving rats were bright, alert and responsive at 7 d with receding toxic signs of botulism. The LD_50_ was calculated from survival outcomes using simple linear regression. The LD_50_, 95% CI and R^2^ values are presented within the figure. (B) Progression of toxic signs in rats challenged with 0.44 ng/kg (2.5 LD_50_) BoNT/A (n = 12). (C) Survival curve for rats from panel B.

**Additional file 2: Figure S2. **Effects of 3,4-DAP on LC/A proteolytic activity in a FRET-based substrate cleavage assay. The FRET-based SNAP-25 substrate was incubated with 100 pM LC/A plus saline vehicle (veh) or 0.01–100 µM 3,4-DAP (n = 5 wells for each condition) for 1 h at 37 C. FRET ratios were measured by exciting reactions at 434 nm and comparing fluorescent emission at 540 nm versus 476 nm (red columns). Comparison of FRET ratios revealed no significant effect of 3,4-DAP on LC/A activity at any concentration (*p* = 0.38). Control experiments (gray columns) include FRET substrate alone (NV), FRET substrate plus 100 µM 3,4-DAP (NV + DAP) to confirm that 3,4-DAP does not alter fluorescent excitation or emission, and 100 nM BoNT/A to achieve full substrate cleavage. 3,4-DAP did not affect fluorescent responses of the substrate (*p* = 0.99), while 100 nM BoNT/A significantly reduced FRET activity (*p* < 0.0001). *ns*, not significant.

**Additional file 3: Video S1. **3,4-DAP-infused rats at 21 d after challenge with 2.5 LD_50_ BoNT/A. Rats were challenged with 2.5 LD_50_ BoNT/A and continuously infused with 3,4-DAP from 1–14 d at 1.5 mg/kg∙h (A-D), 1.0 mg/kg∙h (E–H) or 0.5 mg/kg∙h (I). Representative video recordings of rat activity were collected at 21 d after intoxication. Videos demonstrate normal levels of activity, including grooming, exploratory behaviors and chow consumption.

**Additional file 4: Table S1**. Peak neurological toxic signs in rats following treatment with 3,4-DAP. Healthy rats (n = 4 per group) were evaluated for neurophysiological toxicities following 3,4-DAP administration. Assessments of peripheral neurological effects included gait and salivation. Central nervous effects were evaluated using the Racine scale (Luttjohann et al. 2009). Assessments were conducted at 30 min intervals after single injections, at 30 min after each injection during a treatment series, and at 6 h intervals during continuous infusions. The peak score is reported for each treatment.

**Additional file 5: Table S2.** Details of statistical tests and parameters.

## Data Availability

Not applicable.

## Abbreviations

BoNT: Botulinum neurotoxin
3,4-DAP: 3,4-Diaminopyridine
HC: Heavy chain
LC: Light chain
SNARE: Soluble *N*-ethylmaleimide-sensitive factor attachment protein receptor
Css: Steady-state serum concentrations
FRET: Fluorescence resonance energy transfer
RFU: Relative fluorescence unit
RMP: Resting membrane potential
EPP: Endplate potential
mEPP: Miniature endplate potential
QC: Quantal content
IQR: Interquartile ratio
LEMS: Lambert-Eaton Myasthenic Syndrome
