# Supplementary figures and images for: Antidotal treatment of botulism in rats by continuous infusion with 3,4-diaminopyridine
Source: Mol Med. 2022 Jun 3;28:61. doi: 10.1186/s10020-022-00487-4 (PMC9164507; doi:10.1186/s10020-022-00487-4)

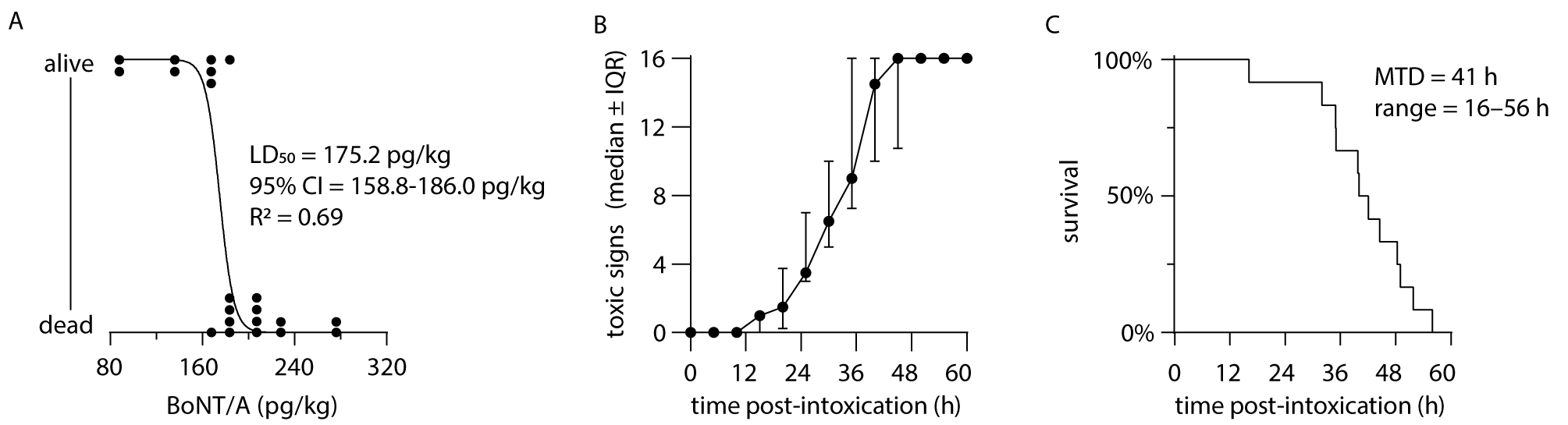

Supplement: Supplementary file 1 — Additional file 1: Figure S1. BoNT/A potency determination and disease progression at 2.5 LD50 in rats. (A) Determination of rat intravenous LD50. Rats were administered 88–276 pg/kg BoNT/A by tail vein injection and monitored for survival at 24 h intervals through 7 d. Surviving rats were bright, alert and responsive at 7 d with receding toxic signs of botulism. The LD50 was calculated from survival outcomes using simple linear regression. The LD50, 95% CI and R2 values are presented within the figure. (B) Progression of toxic signs in rats challenged with 0.44 ng/kg (2.5 LD50) BoNT/A (n = 12). (C) Survival curve for rats from panel B. [file 10020_2022_487_MOESM1_ESM.tif]

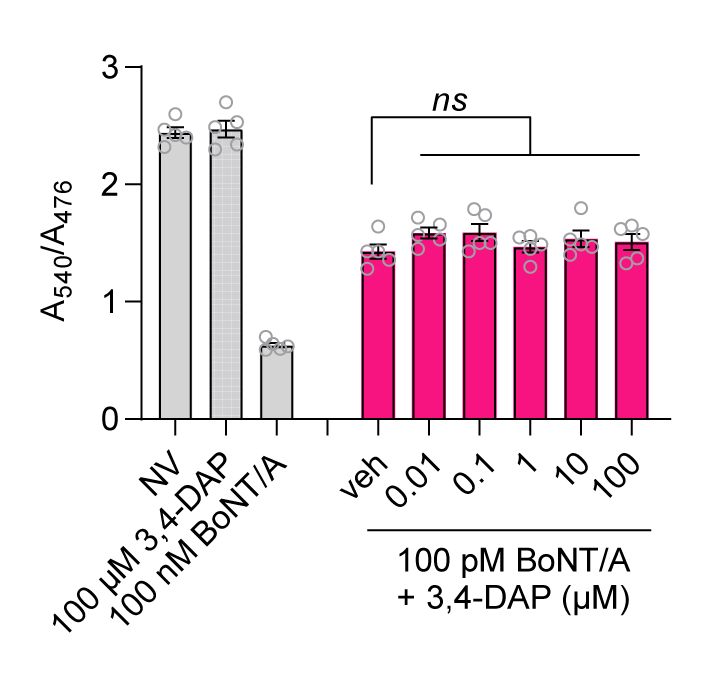

Supplement: Supplementary file 2 — Additional file 2: Figure S2. Effects of 3,4-DAP on LC/A proteolytic activity in a FRET-based substrate cleavage assay. The FRET-based SNAP-25 substrate was incubated with 100 pM LC/A plus saline vehicle (veh) or 0.01–100 µM 3,4-DAP (n = 5 wells for each condition) for 1 h at 37 C. FRET ratios were measured by exciting reactions at 434 nm and comparing fluorescent emission at 540 nm versus 476 nm (red columns). Comparison of FRET ratios revealed no significant effect of 3,4-DAP on LC/A activity at any concentration (p = 0.38). Control experiments (gray columns) include FRET substrate alone (NV), FRET substrate plus 100 µM 3,4-DAP (NV + DAP) to confirm that 3,4-DAP does not alter fluorescent excitation or emission, and 100 nM BoNT/A to achieve full substrate cleavage. 3,4-DAP did not affect fluorescent responses of the substrate (p = 0.99), while 100 nM BoNT/A significantly reduced FRET activity (p < 0.0001). ns, not significant. [file 10020_2022_487_MOESM2_ESM.tif]
