# Supplementary material for: Antidotal treatment of botulism in rats by continuous infusion with 3,4-diaminopyridine
Source: Mol Med. 2022 Jun 3;28:61. doi: 10.1186/s10020-022-00487-4 (PMC9164507; doi:10.1186/s10020-022-00487-4)
Supplement: Supplementary file 4 — Additional file 4: Table S1. Peak neurological toxic signs in rats following treatment with 3,4-DAP. Healthy rats (n = 4 per group) were evaluated for neurophysiological toxicities following 3,4-DAP administration. Assessments of peripheral neurological effects included gait and salivation. Central nervous effects were evaluated using the Racine scale (Luttjohann et al. 2009). Assessments were conducted at 30 min intervals after single injections, at 30 min after each injection during a treatment series, and at 6 h intervals during continuous infusions. The peak score is reported for each treatment. [file 10020_2022_487_MOESM4_ESM.docx]

**Table S1. Peak neurological toxic signs in rats following treatment with 3,4-DAP.**

| treatment route | gait change  (%) | salivation  (%) | behavioral seizure  (%), max Racine score | mortality  (%) |
| --- | --- | --- | --- | --- |
| bolus injection (sc) |  |  |  |  |
| 1 x 1 mg/kg | 0 | 0 | 0, 0 | 0 |
| 1 x 2 mg/kg | 0 | 0 | 0, 0 | 0 |
| 1 x 8 mg/kg | 0 | 0 | 0, 0 | 0 |
| 1 x 16 mg/kg | 50 | 25 | 0, 0 | 0 |
| 15 x 2 mg/kg | 0 | 0 | 0, 0 | 0 |
|  |  |  |  |  |
| infusion (sc) |  |  |  |  |
| 0 mg/kg•h | 0 | 0 | 0, 0 | 0 |
| 0.36 mg/kg•h | 0 | 0 | 0, 0 | 0 |
| 0.72 mg/kg•h | 0 | 0 | 0, 0 | 0 |
| 1.44 mg/kg•h | 0 | 0 | 0, 0 | 0 |
