# Supplementary material for: Antidotal treatment of botulism in rats by continuous infusion with 3,4-diaminopyridine
Source: Mol Med. 2022 Jun 3;28:61. doi: 10.1186/s10020-022-00487-4 (PMC9164507; doi:10.1186/s10020-022-00487-4)
Supplement: Supplementary file 5 — Additional file 5: Table S2. Details of statistical tests and parameters. [file 10020_2022_487_MOESM5_ESM.docx]

**Table S2. Summary of statistical tests**

| **Figure** | **sample size (n);**  **number of studies (ns)** | **compared value** | **primary test** | **primary test result** | **secondary test** | **secondary test result** | **Bonferroni-adjusted significance threshold** |
| --- | --- | --- | --- | --- | --- | --- | --- |
| Figure 1B | vehicle: n=4, ns=1  3,4-DAP: n=2, ns=1 | toxic signs over time | Two-way repeated measures ANOVA | F(11,66)=4.715, ***p*<0.0001** | Sidak’s multiple comparisons test | see figure |  |
| Figure 1C,D | vehicle: n=6; ns=2  3,4-DAP: n=6; ns=2 | toxic signs over time | two-way repeated measures ANOVA | F(22,220)=126.2, ***p*<0.0001** | Sidak’s multiple comparisons test | see figure |  |
|  |  | survival at last treatment | two-sided Fisher’s exact test | vehicle (0/6) vs 3,4-DAP (6/6): ***p*=0.0022** |  |  |  |
|  |  | median survival time | Mantel-Cox log-rank test | vehicle (47.0 h) vs 3,4-DAP (59.8 h): χ^2^=12.1, **p=0.0005** |  |  |  |
| Figure 2 | saline vehicle: n=7; ns=1  0.36 mg/kg•h: n=7; ns=1  0.72 mg/kg•h: n=7; ns=1  1.44 mg/kg•h: n=7; ns=1 | infusion dose-rate vs C_SS_ | simple logistical regression | C_SS_=129.2*IDR; R^2^=0.94 |  |  |  |
|  |  | non-zero slope | simple logistical regression | F=807.4, ***p*<0.0001** |  |  |  |
| Figure 3 | saline vehicle: n=14; ns=5  0.5 mg/kg•h: n=8; ns=3  1.0 mg/kg•h: n=10; ns=4  1.5 mg/kg•h: n=7; ns=3 | 24 h toxic signs | Kruskal-Wallis test | H=3.2, *p*=0.36 |  |  |  |
|  |  | survival proportion at 21 d | Chi-square test | χ^2^=32.8, ***p*<0.0001** | pairwise two-sided Fisher’s exact test | vehicle (0/14) *vs* 0.5 mg/kg•h (1/8): *p*=0.36  vehicle (0/14) *vs* 1.0 mg/kg•h (9/10): ***p*<0.0001**  vehicle (0/14) *vs* 1.5 mg/kg•h (8/8): ***p*<0.0001** | 0.017 |
|  |  | median survival time | Mantel-Cox log-rank test | χ^2^=42.8, ***p*<0.0001** | pairwise Mantel-Cox log-rank test | vehicle (2.5 d) *vs* 0.5 mg/kg•h (4.7 d): ***p*=0.015**  vehicle (2.5 d) *vs* 1.0 mg/kg•h (>50% survival): ***p*<0.0001**  vehicle (2.5 d) *vs* 1.5 mg/kg•h (>50% survival): ***p*<0.0001** | 0.017 |
|  |  | toxic signs over time | two-way repeated measures ANOVA | F(3,36)=60.4, ***p*<0.0001** | Tukey’s multiple comparisons test | vehicle vs 0.5 mg/kg•h: *p*=0.12  vehicle vs 1.0 mg/kg•h: ***p*<0.0001**  vehicle vs 1.5 mg/kg•h: ***p*<0.0001**  0.5 vs 1.0 mg/kg•h: ***p*<0.0001**  0.5 vs 1.5 mg/kg•h: ***p*<0.0001**  1.0 vs 1.5 mg/kg•h: *p*=0.93 |  |
|  |  | weight change over time | two-way repeated measures ANOVA | F=0.42, *p*=0.54 |  |  |  |
| Figure 4 | **n (diaphragms, endplates), ns**  naïve: n=4, 80; ns=2  0.5 mg/kg•h: n=5, 100; ns=2  1.0 mg/kg•h: n=8, 160; ns=2  1.5 mg/kg•h: n=3, 60; ns=2 | EPP success rate | Kruskal-Wallis ANOVA | F(4,384)=81.5, ***p*<0.0001** | Dunn’s multiple comparisons test | see figure |  |
|  |  | QC | one-way ANOVA | F(3,378)=93.9, ***p*<0.0001** | Tukey’s multiple comparisons test | see figure |  |
|  |  | mEPP frequency | one-way ANOVA | F(3,377)=86.2, ***p*<0.0001** | Tukey’s multiple comparisons test | see figure |  |
|  |  | mEPP amplitude | one-way ANOVA | F(3,349)=2.1, *p*=0.10 | Tukey’s multiple comparisons test | see figure |  |
| Figure S1A | 88 pg/kg: n=2; ns=2  136 pg/kg: n=2; ns=2  168 pg/kg: n=4; ns=3  184 pg/kg: n=5; ns=3  208 pg/kg: n=4; ns=2  228 pg/kg: n=2; ns=2  276 pg/kg: n=2; ns=2 | BoNT/A rat LD_50_ | simple linear regression | LD_50_=175.2 (95% CI: 158.8 – 186.0)  Tjur’s R^2^=0.69 |  |  |  |
| Figure S1B,C | 440 pg/kg: n=12; ns=2 | % mortality | descriptive | 100% |  |  |  |
|  |  | median survival time | Kaplan-Meier survival curve | 41.0 h (range: 16-56 h) |  |  |  |
| Figure S2 | n=5 wells per condition  6 3,4-DAP concentration: 0, 0.01, 0.1, 1, 10, 100 µg/mL | FRET ratio | one-way ANOVA with Dunnett’s multiple comparisons test | F(5,24)=1.12, *p*=0.38 |  |  |  |
